# An Improved Long Short-Term Memory Algorithm for Cardiovascular Disease Prediction

**DOI:** 10.3390/diagnostics14030239

**Published:** 2024-01-23

**Authors:** T.K. Revathi, Sathiyabhama Balasubramaniam, Vidhushavarshini Sureshkumar, Seshathiri Dhanasekaran

**Affiliations:** 1 Department of Computer Science and Engineering, Sona College of Technology, Salem 636005, India; revathi.tk@sonatech.ac.in; 2 Department of Computer Science and Engineering, Faculty of Engineering and Technology, SRM Institute of Science and Technology, Vadapalani Campus, Chennai 600026, India; vidhushs@srmist.edu.in; 3 Department of Computer Science, UiT The Arctic University of Norway, 9037 Tromsø, Norway

**Keywords:** cardiovascular disease, long short-term memory, salp swarm algorithm, genetic algorithm, disease prediction model

## Abstract

Cardiovascular diseases, prevalent as leading health concerns, demand early diagnosis for effective risk prevention. Despite numerous diagnostic models, challenges persist in network configuration and performance degradation, impacting model accuracy. In response, this paper introduces the Optimally Configured and Improved Long Short-Term Memory (OCI-LSTM) model as a robust solution. Leveraging the Salp Swarm Algorithm, irrelevant features are systematically eliminated, and the Genetic Algorithm is employed to optimize the LSTM’s network configuration. Validation metrics, including the accuracy, sensitivity, specificity, and F1 score, affirm the model’s efficacy. Comparative analysis with a Deep Neural Network and Deep Belief Network establishes the OCI-LSTM’s superiority, showcasing a notable accuracy increase of 97.11%. These advancements position the OCI-LSTM as a promising model for accurate and efficient early diagnosis of cardiovascular diseases. Future research could explore real-world implementation and further refinement for seamless integration into clinical practice.

## 1. Introduction

In recent times, a surge in fatalities has been linked to cardiovascular disease, with the predominant factor being challenges to the heart’s ability to efficiently pump blood throughout the body, causing disruptions in blood circulation [1]. Among the spectrum of heart-related ailments, cardiovascular disease (CVD) stands out as the most detrimental to human health. Its escalating prevalence has positioned CVD as a leading cause of heightened mortality rates, presenting substantial challenges to global healthcare industries [2]. According to surveys, CVD has accounted for a staggering 4 in 10 fatalities, affecting nearly 17.9 million individuals, with a particularly pronounced impact in Asia [3,4].

Various attributes or features, such as sex, age, fasting blood sugar, chest pain [5], chest pain type, chest pain location, blood sugar level, cigarette habit, depression level, electrocardiogram [6], exercise-induced angina (exang), resting electrocardiographic results, slope, old peak, heart status, poor diet, cholesterol, obesity, family history, alcohol intake, high blood pressure, and physical inactivity [7,8,9,10,11,12,13], have been used in different research studies.

CVD prediction traditionally relies on invasive methods, depending on a patient’s medical history and an analysis report from a medical scientist. Moreover, it is a challenging and costly process. Non-invasive methods, such as clinical decision-making support models using Machine Learning (ML) and Deep Learning (DL) approaches, are instrumental in addressing these issues.

The combination of CNN–LSTM (Convolutional Neural Network–Long Short-Term Memory) methods was used to automatically detect COVID-19. This combination employs three types of X-ray images for disease prediction, with LSTM serving as a classifier to distinguish various COVID-19 cases [14]. It ensures better results for image datasets of various sizes and resolutions [15], effectively addressing the issue of overfitting [16,17].

Deep Learning has made significant contributions in various domains, including medical imaging, disease tracking, protein structure analysis, drug discovery, and assessing virus severity and infectivity to control the COVID-19 outbreak [18,19]. Modern technologies such as Deep Learning, Machine Learning, and Data Science are contributing to the fight against all types of deadly diseases [20].

The primary objective of the proposed OCI-LSTM model is to resolve issues that have been detrimental to the performance of prediction models. The main issue is related to data training, which leads to overfitting and underfitting. Another issue is optimizing the network model’s configuration. The model tends to overfit by learning even from small details in the training data, leading to inadequate results when applied to test data [21,22]. Poor learning on the part of the model results in underfitting, where both training and testing data produce poor results. The core reasons for these issues lie in the inappropriate design of the network model and its configuration, as well as in the presence of irrelevant features. These issues increase both the computational cost and the prediction time for CVD. To address this, the Salp Swarm Algorithm (SSA) is employed to remove noisy or duplicate features, helping to find the optimal features effectively. Furthermore, an improved LSTM is proposed for classification, with the Genetic Algorithm (GA) used to optimize the network configuration. The GA fine-tunes the model by selecting the right time window size, offering an optimal solution and enhancing model performance.

Finally, experiments are conducted, and four performance metrics are considered for model evaluation. The Cleveland dataset from the online UCI repository is used for training and testing, a dataset commonly employed in heart disease research.

## 2. Related Works

In this section, we discuss the usage of various optimization algorithms, classifiers, performance metrics applied, and the results obtained in different research works. Finally, we identified the gaps observed in the related works.

Latha and Jeeva [23] utilized an ensemble approach, combining multiple classifiers and employing bagging and boosting techniques to enhance the accuracy of their prediction model. Tao et al. [24] applied Machine Learning techniques to classify ECG signal recordings, achieving a high accuracy of 94.03%. However, they acknowledged a generalization issue in their work, indicating a need for improvement in extending their model’s applicability beyond the experimental setting.

Arabasadi et al. [25] proposed a hybrid approach integrating the GA with classifiers for predicting coronary arterial disease. Issues such as the suboptimal learning rate and momentum factors contributed to this limitation. Pérez et al. [26] introduced latent Dirichlet allocation for discovering insights from the dataset, while their model was evaluated using qualitative and quantitative measures.

Chatzakis et al. [27] presented a cardiovascular prediction approach based on ECG images and patients’ medical history data, forming a Decision Support System (DSS). However, their focus on maintaining health records limited information about predicting the CVD risk factors. Mohan et al. [28] proposed a hybridized linear-based Machine Learning approach using Random Forest to enhance the accuracy. They achieved 88.4% accuracy on the Cleveland UCI repository but faced challenges due to the absence of restrictions on feature selection.

Ali et al. [29] employed a Deep Belief Network (DBN) for heart disease prediction, optimizing it with the Ruzzo–Tompa feature selection algorithm. Despite achieving an accuracy of 94.61%, time complexity issues arose due to suboptimal feature selection. Wang et al. [30] introduced a Deep Neural Network to address the data imbalance, utilizing a Recurrent Neural Network (RNN). While achieving accuracies ranging from 83.84% to 87.54% on different databases, the model did not determine the optimal size of the time window for hidden layers.

Mirjalili et al. [31] proposed optimization techniques, the SSA and Multi-Objective Salp Swarm Algorithm (MSSA), with the MSSA showing high network convergence. Hsiao et al. [32] utilized a Deep Learning framework for cardiovascular risk prediction, employing autoencoders for feature selection and softmax for classification. However, potential network generalization problems were noted.

Abdeldjouad et al. [33] introduced hybridized approaches, including the MOEFC, Logistic Regression, and AdaBoost, with feature selection using the Wrapper method. The model did not outperform other Machine Learning models. Gers and Schmidhuber [34] proposed an LSTM variant, while Chung et al. [35] introduced the Gated Recurrent Unit (GRU). Altan et al. [36] applied the Hilbert–Huang transform for ECG analysis, but they did not use feature selection.

Hochreiter and Schmidhuber [37] introduced the LSTM as an RNN with long-term memory but faced challenges in terms of the computation volume and time costs. Modifications by healthcare researchers aimed to enhance the LSTM’s performance. Javeed, A. et al. [38] proposed the FWAFE method for feature selection, using ANN and DNN frameworks for heart disease diagnosis. However, the achieved accuracies ranged widely from 50.00% to 91.83%.

Javeed, A. et al. [39] developed a Machine Learning-based diagnostic system for coronary artery disease detection. They conducted a systematic review of heart disease prediction methods but did not propose new work, focusing on comparing previous methods. Al Bataineh, A. and Manacek, S. [40] developed and compared Machine Learning-based systems for heart disease prediction using the Cleveland Heart Disease dataset. Their alternative MLP training technique and PSO algorithm achieved an accuracy of 84.61%.

Hassan, C.A. et al. [41] explored Machine Learning techniques for coronary heart disease prediction, using 11 classifiers. Random Forest outperformed the others with a 96% accuracy level. Kurian, N.S. et al. [42] conducted a comparative analysis of Machine Learning classifiers for heart disease prediction with minimal attributes. They evaluated Nearest Neighbor, Gradient Boosting, Support Vector Machine, Naive Bayes, Logistic Regression, and Random Forest, identifying attribute correlation and effectiveness.

Rana, M. et al. [43] employed common Machine Learning methods for heart disease prediction, using the Kaggle dataset. They provided a comparative analysis of the SVM, Naïve Bayes, Random Forest, Decision tree, and K-Nearest Neighbor, emphasizing their utility in classification tasks. Islam, M. et al. [44] presented five supervised Machine Learning techniques for the Wisconsin Breast Cancer dataset, with ANNs achieving the highest accuracy, precision, and F1 score.

Hasan, M.K. et al. [45] developed a mathematical model for breast cancer detection using symbolic regression. They achieved successful detection with minimal errors using the UCI Machine Learning repository dataset. Ayon, S.I. and Islam, M.M. [46] developed a Deep Neural Network model for diabetes diagnosis using the PID dataset, demonstrating high accuracy and performance through cross-validation.

Haque, M.R. et al. [47] presented an expert scheme for liver disorder classification using RFs and ANNs. They achieved accuracy rates of 80% and 85.29% for RFs and ANNs, respectively. Ayon, S.I. et al. [48] compared computational intelligence techniques for coronary artery heart disease prediction, finding that DNN achieved the highest accuracy of 98.15%.

This literature review exposes the limitations of existing methodologies for predicting heart disease risk factors, highlighting challenges in effectively mitigating overfitting and underfitting, employing time-intensive optimization techniques, and relying on traditional diagnostic tools such as ECG. These research gaps are summarized in Table 1. To address these challenges, this paper introduces the OCI-LSTM as a solution to prevent cardiovascular disease (CVD). The integration of the LSTM with the GA is intended to enhance the predictive capabilities. The OCI-LSTM is applied to the well-established Cleveland Heart Disease dataset, addressing overfitting through optimal feature selection. The model also tackles network configuration challenges by randomly determining the number of suitable layers and hyperparameters. The OCI-LSTM is specifically designed to overcome the identified issues and elevate the overall model performance.

## 3. Proposed OCI-LSTM Approach

We present a new model aimed at improving the accuracy of CVD prediction while addressing network generalization problems, specifically overfitting and underfitting. Additionally, it deals with configuration- and optimization-related issues, such as determining the optimal network configuration. The process begins with preprocessing using the min–max scaling algorithm. Then, the best attributes are selected through the SSA. Subsequently, these optimized features are fed into the OCI-LSTM, which effectively resolves the mentioned issues and enhances the prediction accuracy level.

### 3.1. Min–Max Scaling for Feature Normalization

In this phase, the preprocessing involves normalizing missing values and irrelevant data using the min–max scaling method. The dataset encompasses various attributes, such as age, patient gender, type of chest pain, resting and fasting blood pressure, maximum heart rate, slope of the ST segment, exercise-induced angina, number of primary vessels, etc. Regular and systematic monitoring aids in comprehensive data collection for the repository. However, challenges arise due to missing data values, patient interruptions, and technical faults during the collection process, impacting disease analysis [49,50,51].

The collected data, containing missing values and irrelevant information, pose a potential hindrance to the model’s performance. Therefore, it is crucial to address these issues by applying the standard scaling method to the entire dataset and evaluating the distributed data outcomes [52,53,54]. Following this process, noisy and unnecessary data are eliminated, retaining only pertinent information.

Upon examination, it is discovered that among the 330 instances in the dataset, 6 instances contain missing values. To ensure standardized results for easy interpretation during model training, the normalization process is applied, and the standardized outcome is calculated using Equation (1).

(1) $$Standardised\;outcome=\frac{X_{value}-mean}{sd}\times 100$$

where $X_{value}$ is the maximum of the heart data value, and Standard Deviation (SD) is the standard deviation.

All the six instances are handled effectively with the use of Equation (1). The dataset mean is calculated using Equation (2).

(2) $$mean=\frac{\sum_{i=1}^{n}x}{n}$$

After calculating the mean, the SD is computed using the Equation (3).

(3) $$Standard\;Deviation=\sqrt{\frac{1}{N}\sum_{i=1}^{n}{\left(x_{i}-mean\right)}^{2}}$$

where N is the total number of samples considered for calculating the SD. Given that the Cleveland dataset contains features with different ranges and magnitudes, we normalize the entire dataset, particularly the nominal features. However, the categorical features are not suitable for the scaling process. Therefore, the min–max approach is used to adjust the values to a range of 0–1. This adjustment aids the model in interpreting the data easily during the training phase.

The data normalization is performed as follows:

(4) $$N^{\prime }=\frac{X_{value}-X_{min\;\_value}}{X_{max\;\_value}-X_{min\;\_value}}$$

In Equation (4), $N^{\prime }$ is denoted as normalized data, $X_{value}$ is noted as a particular data of any instance, $X_{min\;\_value}$ is represented as the minimum value of the whole dataset, and $X_{max\_value}$ is represented as the maximum value of the whole dataset.

This paper extends its analysis by estimating various parameters, such as the variance, minimum, maximum, correlation, and energy, to mitigate the risk factors associated with CVD during disease prediction. In the initial step, features that seem to provide no value are substituted with new ones. The dataset often contains extensive patient information, and while some features are relevant for disease prediction, others may be deemed irrelevant, potentially leading to overfitting. To address this, the paper incorporates feature set reduction along with optimization to enhance the disease recognition process. The Salp Swarm approach is employed to obtain the most essential optimized features from the original dataset, as detailed in the following section.

### 3.2. Salp Swarm for Finding an Optimal Subset Feature

In this section, the focus is on selecting the most appropriate and useful features for the model to predict disease with greater efficiency. The SSA is employed for the purpose of attribute selection, enhancing the model’s learning process by eliminating unwanted attributes.

The SSA leverages the swarming mechanism observed in salps, a type of marine organism, to randomly select a population. In the sea, a salp chain, known as a salp swarm, is formed, with the leader salp positioned at the front end and the remaining salps as swarm followers. Salp positions are denoted in an n-dimensional search location, where ‘n’ represents the total count of identifiers in a given problem. The feature optimization process encompasses three steps: 1. initializing the population, 2. updating the leader’s position, and 3. updating the follower’s position. These steps reflect the clustering process of a salp swarm. The subsections below provide a detailed discussion of the working principle of the SSA.

#### 3.2.1. Initializing Population

The population initialization is carried out in the S×D Euclidean workspace, where *S* is the swarm scale and *D* is the dimension in space. Consider the available food in the space to be *fd* and it is assigned as *fd* = [${fd}_{1}$, ${fd}_{2}$, …, ${fd}_{d}$]*^T^* where the position of each salp can be denoted as $P_{n}={\left[P_{n,1},P_{n,2},\ldots ,P_{n,D}\right]}^{T}$, where *n* = 1, 2, ...., *N*. The upper and lower bound is denoted as *U_b_*, *L_b_*. The upper bound is said as $U_{b}\;$= ${\left[U_{b1},U_{b2},\ldots ,U_{bD}\right]}^{T}$ and the lower bound is represented as $L_{b\;}$= ${\left[L_{b1},L_{b2},\ldots ,L_{bD}\right]}^{T}$.

The random initialization of the population is computed using Equation (5).

(5) $$X_{S\times D}=rand\left(S\times D\right)\times \left(U_{b}-L_{b}\right)+L_{b}\times ones(S\times D)$$

The leader and followers state of the population in the dth dimension are $x_{1,d}$ and $x_{k,d}$ where, k=2,3,…,N.

#### 3.2.2. Updating Leader Position

In a salp swarm, the leader is responsible for finding food in the space. It must also guide the entire group to move in search of food. It is essential to update the leader’s position, which is achieved using Equation (6).

(6) $$x_{1,d}={fd}_{d}+r_{1}\left(\left(U_{bd}-L_{bd}\right)r_{2}+L_{bd}\right)$$

where $r_{1}$ and $r_{2}$ are random numbers within the interval range [0,1]. The leader’s movement, searching ability, and individual population diversity are randomly enhanced by the parameters mentioned in Equation (6). In all meta-heuristic approaches, there is a key parameter known as $r_{1}$, as defined in Equation (6). This parameter is also referred to as the convergence factor. During the iteration process, this parameter balances the trade-off between exploitation and exploration. If $r_{1}$ is greater than 1, the algorithm performs global exploration. If $r_{1}$ is less than 1, it focuses on local exploration to find an accurate estimation value. The value of $r_{1}$ should fall within the range of 2 to 0 for the initial iteration of the algorithm to conduct global search and subsequently improve the accuracy of the following iterations. The convergence factor is calculated using Equation (7).

(7) $$r_{1}=2e^{-{\left(\frac{4i}{i_{max}}\right)}^{2}}$$

where i represents the current iteration and $i_{max}$ denotes the total number of iterations.

#### 3.2.3. Updating Follower Position

In the SSA, the followers adopt a series of chain movements rather than random movements. To determine the followers’ movement, certain important aspects need to be considered, including the followers’ initial position, speed of motion, and acceleration. Newton’s law of motion is followed to calculate the motion distance, and it is computed using Equation (8).

(8) $$Motion\;Distance=\frac{1}{2}\alpha i^{2}+s_{0}i$$

where i is the iteration during the optimization process, i = 1 when there is discrepancies happens between iterations, $s_{0}$ is the speed of the followers, and it becomes 0 at the first iteration, and α is the followers’ acceleration, as calculated between the first and last iterations.

The followers’ acceleration is calculated using Equation (9). Always the followers follow the predecessor salp.

(9) $$\alpha =\frac{\left(s_{final}-s_{0}\right)}{t}$$

So, the salp’s movement speed can be determined using Equation (10).

(10) $$s_{final}=\left(x_{k-1,d}^{i}-x_{k,d}^{i}\right)/t$$

where t = 1; $s_{0}=0$; hence, the Motion Distance is assigned as Equation (11).

(11) $$Motion\;Distance=\frac{1}{2}\left(x_{k-1,d}^{i}-x_{k,d}^{i}\right)$$

The follower position is updated with the help of Equation (12).

(12) $$x_{k,d}^{i+1}=x_{k,d}^{i}+Motion\;Distance=\frac{1}{2}\left(x_{k,d}^{i}+x_{k-1,d}^{i}\right)$$

where $x_{k,d}^{i}$ is the dth dimensional kth follower in the ith iteration and $x_{k,d}^{i+1}$ is representing the followers’ position in the (i+1) th iteration. Algorithm 1 describes the flow of the SSA.
**Algorithm 1.** Salp Swarm Algorithm1. Initialization:     Salp swarm random population generation $X_{i}$ where *i* = 1,2,3, . . . ., *n*2. Determine each salp’s fitness value.3. Assign $X^{*}$ as one of the best searching agents.4. While the end condition has not arrived5. Update the convergence factor $r_{1}$ by Equation (7)6. For each and every salp7. If (n = = 1)8. Update leader salp’s position, $x_{1,d}$ using Equation (6)9. Else10. Update the follower salps’ position, $x_{k,d}^{i+1}$ using Equation (12)11. End if12. End for13. Estimate each salp’s fitness value using Equation (14).14. Update the $X^{*}$ with its finest solution.15. End while16. Return $X^{*}$ along with its best fitness value. 

### 3.3. Genetic Algorithm for Optimization

The GA draws inspiration from natural evolution and is categorized as a meta-heuristic approach [55]. In essence, the GA employs fundamental principles of genetics and evolution, incorporating crossover and mutation. Optimal solutions in the GA are derived by selecting the fittest individuals from each generation. The core process of the GA involves various operators to choose qualified members of the current generation [56,57].

The selection operator facilitates individuals’ involvement in determining the next generation based on their fitness. It shapes the subsequent population level by evaluating the compatibility of the current generation. Stochastic Universal Sampling (SUS) and the Roulette Wheel (RW) stand out as commonly used selection operators in the GA [58]. The RW calculates the selection probability for everyone separately. Equation (13) computes the proportionate fitness selection of an individual:

(13) $${\left(Probability\right)}_{i}=\frac{{\left(fitness\right)}_{i}}{\sum_{k=1}^{N}{\left(fitness\right)}_{k}}$$

where ${\left(fitness\right)}_{i}$ is denoted as the fitness of an individual i, ${\left(Probability\right)}_{i}$ is the probability of an individual, and N represents total individuals involved in the population.

Substitution Operator: This operator facilitates the transfer of members from one generation to the next and plays a crucial role in the propagation process:

(14) $${\left(fitness\right)}_{i}=w1\;\times \;Age+w2\times restecg+w3\times maxhr\ldots ..\;\;$$

where w1, w2, w3… are weights assigned to different health indicators.

Recombination Operator: The recombination operator substitutes substrings of two different members from the same generation using the concept of intersection. Common approaches for the recombination operator include single-point, two-point, and uniform crossovers [59].

Mutation Operator: Responsible for changing the genes of members of the current generation to create the next generation. Various methods are available for performing mutation, including uniform, non-uniform, boundary, and Gaussian mutations [60]. The Gaussian operator is commonly used among these methods, adding random values to the selected gene from a normal distribution. Consider if $x\in \left[u,v\right],$ a chosen gene is used for performing the mutation process. Then, $x^{\prime }$ is calculated as given in Equation (15).

(15) $$x^{\prime }=min\left(max\left(N\left(x,\varphi \right),u\right),v\right)$$

where $N\left(x,\varphi \right)\;$represents the mutation operation, and φ mutation rate depends on the time interval.

### 3.4. OCI-LSTM Model

**Figure 1:**
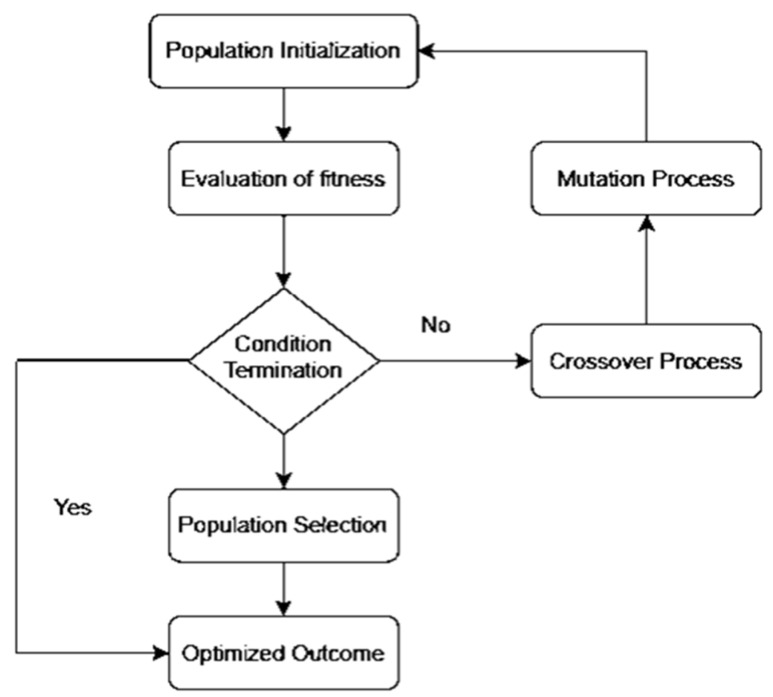
Genetic Algorithm-based network configuration process.

We propose the OCI-LSTM model, integrating it with the Genetic Algorithm (GA) to select the optimized time window for the LSTM units. The LSTM offers a significant advantage by enhancing the model’s performance through the utilization of information from past events to determine the suitable time window. The selection of an appropriate window size is crucial; if too small, the network may overlook essential information, and if too large, the model may become overfitted with training data. Figure 1 illustrates the GA-based network configuration process.

The OCI-LSTM model consists of two phases. In the first phase, parameters are appropriately set. The network includes an input layer and two hidden layers. The GA ensures that the hidden layers contain the optimal number of hidden neurons. Two activation functions are implemented in the OCI-LSTM network: the sigmoid function in the input and hidden nodes to scale input values to the range of −1 to 1, and the linear function for the output nodes, given the problem’s nature in predicting CVD. Initially, the network weights receive random values, later adjusted using the Adam optimizer, known for its computational efficiency [61]. The evolutionary-based search algorithm, the GA, is employed to determine the optimal window size and explore the architectural factors of the OCI-LSTM network.

**Figure 2:**
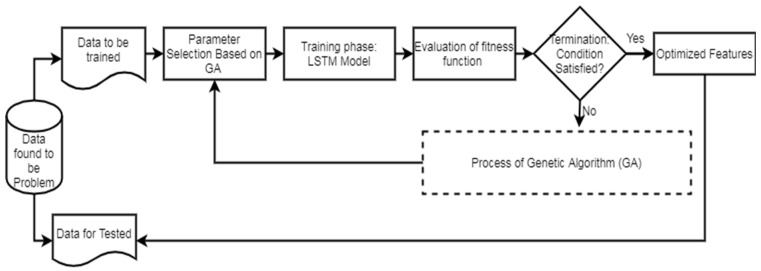
LSTM workflow diagram.

Figure 2 illustrates the workflow of the LSTM. In the second phase of the network model, the evaluation of the GA fitness is conducted. Different LSTM units are utilized in the hidden layers, and various window sizes are applied to the OCI-LSTM for this evaluation. The populations, initially assigned arbitrary values, undergo an initialization process before exploring the two-dimensional space using the operators.

The chromosomes in this work are encoded as binary bits, representing the time window’s size and the LSTM cell counts. Genetic operators then search for the best solution, evaluating these solutions using a fitness method. The Mean Squared Error (MSE) is employed as the fitness function in this work. The smallest MSE value returned by the architectural factors is considered the optimal solution. If the termination condition is satisfied, the derived optimal solution or the nearest optimal solution is applied to the OCI-LSTM model. If not, the entire genetic operations are repeated until the condition is met. Parameters such as the mutation, crossover, and population size are adjusted during experimentation to enhance the model’s fitness and improve the results. In this experiment, the OCI-LSTM uses a crossover parameter value of 0.7 and a mutation rate of 0.15, running for a total of 10 generations to meet the termination criteria.

**Figure 3:**
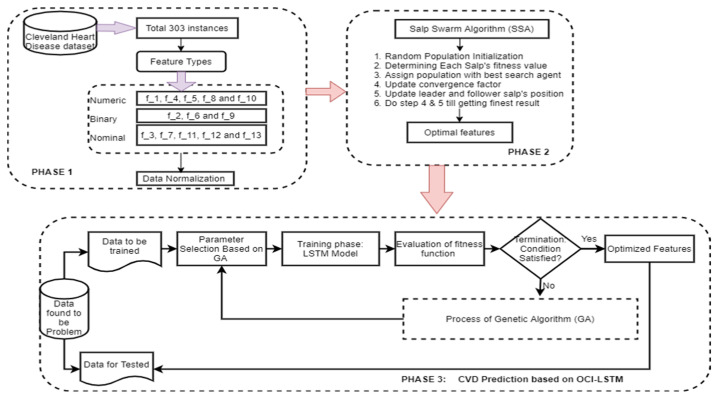
Proposed OCI-LSTM using the Salp Swarm and GA.

**Figure 4:**
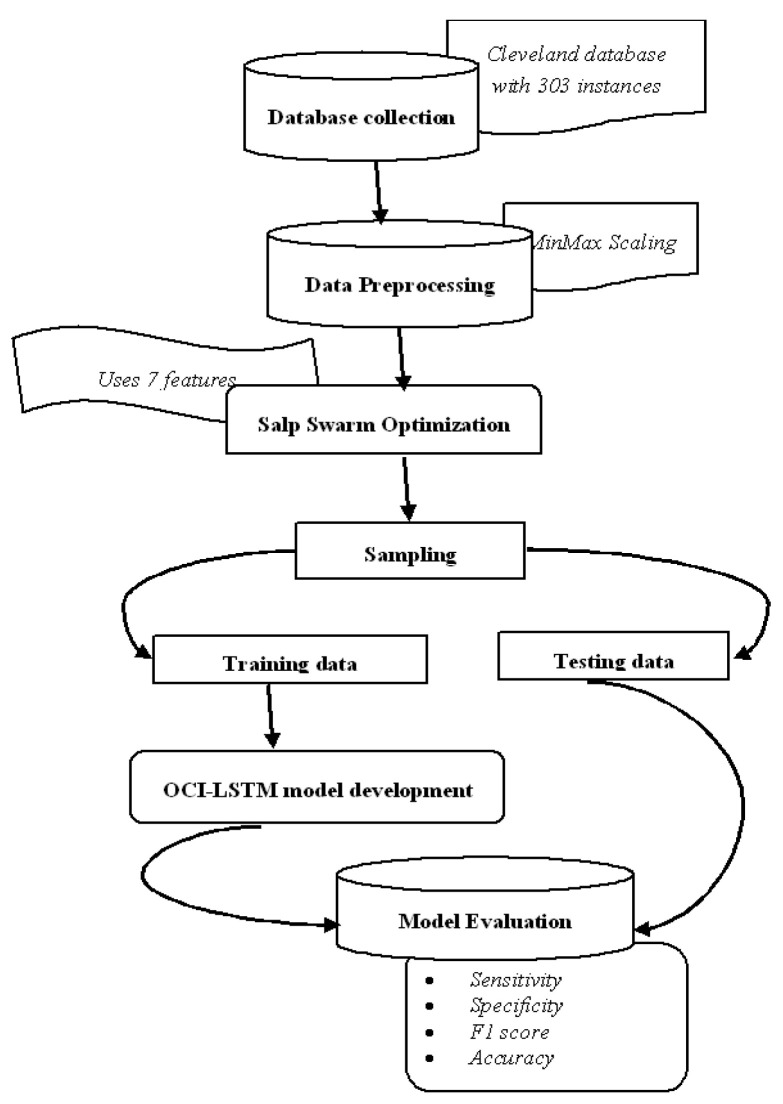
Framework diagram.

Figure 3 illustrates the proposed OCI-LSTM using the Salp Swarm and GA, while Figure 4 provides an overview of the entire framework.

We utilize the publicly available Cleveland dataset from the online UCI repositories [62]. The dataset comprises a total of 76 attributes or features. However, only 13 out of the 76 attributes are commonly used by most researchers for diagnosing heart disease. The dataset consists of 303 instances, with 6 instances having missing values. In the preprocessing phase of the proposed OCI-LSTM process, we begin by removing the outliers from the dataset. As a result, we eliminate 6 instances, leaving us with 297 instances to work with. Table 2 provides a neat sketch of 13 features from the Cleveland dataset.

The process of selecting the most relevant and necessary features using the SSA is crucial for enhancing the model’s performance. The seven selected attributes (f_1, f_7, f_8, f_9, f_10, f_12, and f_13) are considered optimal features based on the SSA. Each of these attributes plays a significant role in predicting CVD in the Cleveland Heart Disease dataset. The clinical significance of all these attributes is provided below

f_1 (Age): Age is a well-established risk factor for CVD. The likelihood of developing cardiovascular issues often increases with age, making it a crucial attribute for prediction models.

f_7 (Serum Cholesterol): Elevated cholesterol levels are associated with an increased risk of heart disease. Monitoring serum cholesterol levels helps in assessing cardiovascular health.

f_8 (Fasting Blood Sugar): High fasting blood sugar levels can indicate diabetes, which is a risk factor for CVD. Monitoring blood sugar is essential for predicting and managing cardiovascular risk.

f_9 (Resting Electrocardiographic Results): Resting electrocardiographic results provide insights into the heart’s electrical activity. Certain patterns or abnormalities in ECG can indicate potential heart issues.

f_10 (Maximum Heart Rate): The maximum heart rate during exercise is a valuable indicator. Abnormalities or deviations from the expected maximum heart rate can signal cardiovascular problems.

f_12 (Exercise-Induced Angina): The presence of angina (chest pain) during exercise is a significant symptom of coronary artery disease. This attribute helps in identifying individuals with exercise-related cardiovascular issues.

f_13 (Old Peak): The ‘Old Peak’ attribute refers to the depression induced by exercise relative to rest. This can be indicative of stress on the heart during physical activity, providing valuable information for CVD prediction.

These selected attributes collectively provide essential information about the patient’s age, biochemical profile, cardiac function at rest and during exercise, and presence of symptoms, which are crucial factors for predicting the risk of cardiovascular disease. The SSA, by optimizing the feature selection process, ensures that the chosen attributes contribute significantly to the model’s predictive accuracy while mitigating overfitting and enhancing overall performance.

The temporal aspects of the data using the proposed network comprise the information about multiple visits of each patient, the time interval between every visit, the target variable’s presence or absence of heart disease, and the number of steps that (visits or intervals) the LSTM network should consider when making predictions.

The class label in the dataset is a multi-class variable that ranges from 0 to 4. Here, the value 0 represents the absence of CVD, while the values from 1 to 4 represent various stages of CVD presence. In this study, we follow the approach outlined in [63] to convert the multi-class label into a binary class label, where label 0 indicates the absence of CVD and label 1 denotes the presence of CVD.

Upon applying this transformation, it is determined that out of the 297 instances in the dataset, 164 correspond to healthy subjects/patients, as indicated by label 0 (absence of CVD).

The cost function of a GA–SSA algorithm is represented in the Equation (16).

(16) $$cost\left(x\right)=w_{1}\times f_{GA}\left(x\right)+w_{2}\times f_{SSA}\left(x\right)\;$$

where x represents the solution, which may include genetic parameters and positions of salps, $f_{GA}\left(x\right)\;$is the objective function or cost associated with the Genetic Algorithm component, ${\;f}_{SSA}\left(x\right)\;$is the objective function or cost associated with the Salp Swarm Algorithm component, and $w_{1}\;$and $w_{2}\;$are weights that control the contribution of each algorithm to the overall cost. The weights $w_{1}$ and $w_{2}$ could be used to balance the influence of the Genetic Algorithm and Salp Swarm Algorithm component on the optimization process.

The minimization of the objective function could be:

$$f_{GA}\left(x\right)=Minimize(x)$$

$$f_{SSA}\left(x\right)=Minimize(x)$$

## 4. Results and Discussion

This work makes several key contributions:

(i) Introduction of the OCI-LSTM: A novel approach, the OCI-LSTM, is proposed to enhance CVD prediction by effectively mitigating both overfitting and underfitting. The method involves the selection of a pertinent feature subset from various optimization algorithms, with the Salp Swarm Algorithm demonstrating superior performance in addressing generalization issues.
(ii) Network Configuration Resolution: The OCI-LSTM model resolves network configuration challenges by identifying temporal patterns, such as the optimal time window size, and determining the finite LSTM units using the GA. The integration of a local search with the GA enhances the model iteratively, ensuring the GA discovers the optimal hidden layers for the LSTM, resulting in the finest OCI-LSTM design.
(iii) Comparative Analysis: The OCI-LSTM is rigorously analyzed by comparing it with conventional models, Deep Neural Network (DNN), and Deep Belief Network (DBN). The results showcase the highest accuracy rate and optimal convergence when compared to the models.
(iv) Outstanding Results with Limited Data: Remarkably, the proposed OCI-LSTM achieves outstanding results using a small volume of the Cleveland Heart Disease dataset and a minimal set of parameters. This highlights the model’s efficiency and effectiveness in CVD prediction, emphasizing its potential for practical implementation in real-world scenarios.

The dataset underwent two-way partitioning into training and testing sets. The training set facilitated model training and the testing set was used to evaluate the classifier’s performance [64,65]. Each partition served as a testing fold to ensure comprehensive testing and training over 10 folds. The performance evaluation utilized key metrics, including the accuracy, sensitivity, precision, and F1 score. These metrics were calculated using the formulas provided in Equations (17)–(20), respectively [66,67,68,69]. The results, averaged over the 10 folds, contributed to a comprehensive assessment of the OCI-LSTM model’s performance.

(17) Accuracy=((TN+ TP)) ÷ ((TN+ TP+ FN+FP))

(18) Sensitivity=TP ÷ (TP+FN)

(19) Precision=TP ÷ (TP+FP)

(20) F1-score=(2× precision× sensitivity) ÷ (precision+sensitivity)

where TP is the True Positives, TN is the True Negatives, FP is the False Positives, and FN is the False Negatives.

The experiment commences by partitioning the entire set of instances in the Cleveland dataset into two subsets. Out of the total 297 instances, 207 instances are allocated to the training set, while the remaining 90 instances are designated for testing the model. The architecture details of the DBN and DNN models are illustrated in Table 3. The distribution of instances for both the training and testing sets is detailed in Table 4. Table 5 is presented, which provides a comparative analysis of the results of all three models.

The comparative analysis presented in Table 5 indicates a variation in the performance of the OCI-LSTM and the other two network models. The results obtained for all four metrics of the OCI-LSTM are superior when compared to the DNN and DBN with optimal features.

**Figure 5:**
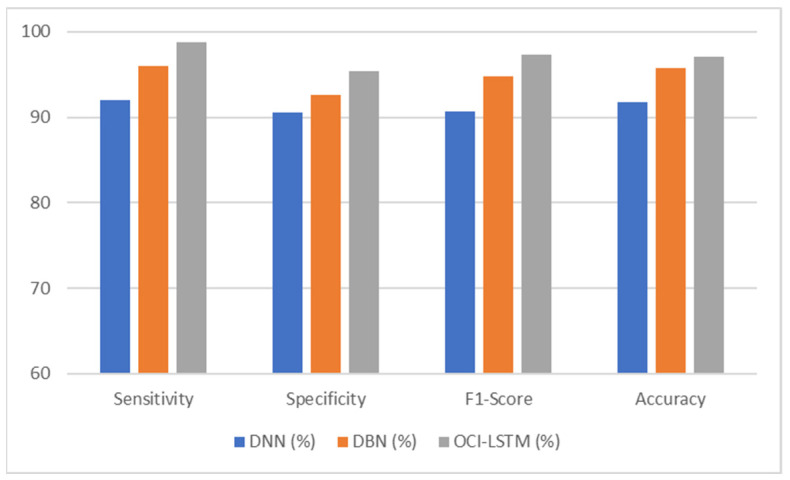
Performance comparison of the proposed model with other existing models.

From Table 6, it is evident that using the SSA, the classification accuracy of the OCI-LSTM has improved when compared with other famous feature optimization algorithms. Figure 5 illustrates the comparison of the proposed model with other existing models.

**Figure 6:**
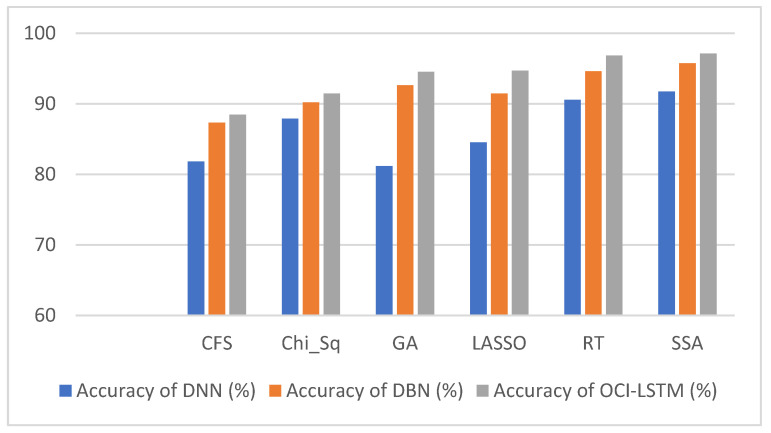
Results comparison of the OCI-LSTM, DNN and DBN with various famous optimization algorithms.

Figure 6 illustrates the comparison of the results using various well-known optimization algorithms applied to the OCI-LSTM, DNN, and DBN. The OCI-LSTM model demonstrates superior performance through rigorous experimentation and comparison with other models and techniques.

The OCI-LSTM starts with a feature optimization step using the SSA. This strategic feature selection process aims to enhance the model performance by identifying the most relevant attributes. Out of thirteen features, seven optimized features (f_3, f_7, f_8, f_9, f_10, f_12, and f_13) are selected based on their strong correlations with the target variable, providing crucial insights for predicting cardiovascular disease. The SSA, employed in feature optimization, is designed to prevent network generalization issues, such as overfitting. Overfitting occurs when a model learns from noise in the training data, leading to poor generalization on unseen data. By selecting the most suitable features, the OCI-LSTM mitigates the risk of overfitting, ensuring robust performance on both training and testing sets.

The efficacy of the OCI-LSTM is evaluated through three comprehensive experiments. In the first experiment, the OCI-LSTM is applied to the selected optimal features. In the second experiment, the same optimal features are used in the DNN and Deep Belief Network (DBN) models, and the results are recorded for comparison. The third experiment involves feeding the OCI-LSTM, DBN, and DNN with the original dataset containing all features. Additionally, the OCI-LSTM is tested with other feature selection methods. These experiments provide a thorough analysis, highlighting the OCI-LSTM’s effectiveness in CVD prediction. The OCI-LSTM is further enhanced by integrating the Genetic Algorithm (GA) for optimizing the time window size for the LSTM units. This integration ensures that the model adapts to the temporal aspects of the data, striking a balance between capturing essential information and avoiding overfitting. The GA-based network configuration process contributes to the model’s overall efficiency.

The OCI-LSTM, particularly when combined with the Salp Swarm and GA, consistently outperforms the other classifiers and conventional techniques. Comparative evaluations against the DNN, DBN, and models utilizing all the features showcase the superior predictive power of the OCI-LSTM. The careful selection of optimal features and the incorporation of evolutionary-based algorithms contribute to its remarkable performance. In summary, the OCI-LSTM model stands out by addressing overfitting, optimizing feature selection, and integrating the GA for improved temporal modeling. The experimental results and thorough comparisons emphasize its effectiveness in predicting cardiovascular disease.

The accuracy of the OCI-LSTM is achieved through a combination of effective feature subset selection, resolution of network configuration challenges, iterative model enhancement through optimization algorithms, and rigorous comparative analysis with other models. The model’s ability to perform well with limited data further underscores its potential for practical implementation in real-world scenarios.

The OCI-LSTM consistently outperforms the DNN and DBN across all metrics, sensitivity, specificity, F1 score, and accuracy, showcasing its superior performance in CVD prediction. The higher sensitivity and specificity of the OCI-LSTM suggest that it is effective in minimizing both false positives and false negatives, making it a robust model for CVD prediction. The high F1 score indicates a good balance between precision and recall in the OCI-LSTM, reinforcing its effectiveness in capturing true positive cases while avoiding false positives and false negatives. Overall, based on these metrics, the OCI-LSTM appears to be a promising model for CVD prediction with a high level of accuracy and reliability.

The OCI-LSTM’s superior performance in CVD prediction, driven by effective feature selection and optimized network configuration, empowers medical practitioners with a reliable tool. The model’s high sensitivity and specificity ensure accurate identification concerning both positive and negative cases, minimizing errors in diagnosis. Its ability to maintain a balanced F1 score underscores its precision and recall, aiding practitioners in capturing true positive cases while avoiding false positives and negatives. The OCI-LSTM’s efficiency enhances practical applicability, providing medical professionals with a robust and accurate predictive tool for timely intervention and personalized patient care in real-world scenarios.

## 5. Conclusions

The Optimally Configured and Improved Long Short-Term Memory (OCI-LSTM) model, integrating feature selection through the Salp Swarm Algorithm (SSA) and network configuration optimization via the Genetic Algorithm (GA), adeptly tackles network generalization challenges like overfitting and underfitting. Through comparative analyses involving the DNN and DBN models, utilizing both the complete feature set and the optimized subset, the OCI-LSTM consistently emerges as the superior performer in terms of accuracy. Rigorous statistical examinations and evaluations underscore the significance of the OCI-LSTM compared to its counterparts. Achieving an impressive accuracy of 97.11%, the OCI-LSTM holds substantial promise in supporting medical professionals in making informed decisions for cardiovascular disease prediction. Furthermore, it involves collaboration with domain experts to validate the significance of the chosen subset of features in the context of cardiovascular health. Fine-tuning hyperparameters, such as the learning rates and dropout rates, may also contribute to enhancing the OCI-LSTM’s robustness and generalization across diverse datasets, and a user-friendly interface and integration into existing healthcare systems could facilitate seamless adoption by medical professionals, promoting its practical utility in real-world clinical settings.

## Figures and Tables

**Table 1:** Research gaps.

Author	Classification Technique	Gap	Accuracy (%)
Latha and Jeeva [23]	Naïve Bayes (NB), C 4.5, Bayes Net, Multilayer Perceptron (MLP), Random Forest and PART	The decline in accuracy is attributed to the absence of an appropriate feature selection algorithm.	85.48
Tao et al. [24]	XG Boost, K-Nearest Neighbor and Support Vector Machine (SVM)	The generalization issue remains in this research work.	94.03
Arabasadi et al. [25]	Roulette Wheel method	The learning rate and momentum factors have not been optimized to the desired level.	78
Pérez et al. [26]	Decision Support System	The model’s overall performance has been compromised due to the constrained search space for dimensionality reduction.	91.6
Chatzakis et al. [27]	Decision Support System	Diagnosing CVD is challenging because the authors have developed only a Decision Support System (DSS) for maintaining health records and have not provided sufficient details about their prediction and the classification of cardiovascular risk factors.	92.3
Mohan et al. [28]	SVM with an Apriori algorithm	The model exhibits no constraints on feature selection, leading to a classification error of up to 11.6%.	88.4
Ali et al. [29]	Deep Belief Network	Time complexity issues arise in the model due to inadequate feature selection.	94.61
Javeed, A. et al. [38]	FWAFE-ANN and FWAFE-DNN	The attained accuracies, ranging from 50.00% to 91.83%, are notably on the lower side.	50.00–91.83
Al Bataineh, A. and Manacek, S. [40]	Multilayer Perceptron	The accuracy obtained reaches a maximum of 84.61%.	84.61

**Table 2:** Brief sketch of 13 features from the Cleveland dataset.

S. No	Sign	Name	Data Type	Description	Range
1	f_1	Age	numeric	Age of subject in years	Between 29 to 77
2	f_2	Sex	Binary	Gender of subject	1—male 0—female
3	f_3	cptype	nominal	Chest pain type	1—typical angina 2—atypical angina 3—non-anginal pain 4—asymptomatic
4	f_4	restbp	numeric	Resting blood pressure	[94:200]
5	f_5	Ser_chol	numeric	Serum cholesterol	[126:564]
6	f_6	fastbp	Binary	Fasting blood sugar	0-false 1-true
7	f_7	restecg	nominal	Resting electrocardiographic	0—normal 1—Abnormal ST-T wave 2— likely/exactly to have left ventricular hypertrophy
8	f_8	maxhr	numeric	Maximum heart rate	[71:202]
9	f_9	exerir	Binary	Exercise-induced angina	0—no 1—yes
10	f_10	Op	numeric	ST depression	[0:6.2]
11	f_11	slopeST	nominal	Slope of ST segment	1—upslope 2—flat 3—downslope
12	f_12	numvesl	nominal	Number of major vessels	0 to 3
13	f_13	Thal	nominal	Thalassemia or defect type	3—normal 6—fixed defect 7—reversable defect

**Table 3:** Architecture details of DBN and DNN models.

Model	Layer Type	Units	Output Size	Activation Function	No. of Trainable Parameters
DBN	Input	-	(None,13)	-	-
	Hidden Layer 1	50	-	Sigmoid	700
	Output	1	(None,1)	Sigmoid	51
DNN	Input	-	(None,13)	-	-
	Hidden Layer 1	100	-	ReLU	200
	Hidden Layer 2	50	-	ReLU	220
	Output	1	(None,1)	Sigmoid	31

**Table 4:** Summary of the training and testing dataset.

S. No	Training Instances	Testing Instances	Total Instances
1	207	90	297

**Table 5:** Overall performance of the models.

Metrics	DNN (%)	DBN (%)	OCI-LSTM (%)
Sensitivity	91.95	96.03	98.78
Specificity	90.54	92.65	95.37
F1 Score	90.73	94.77	97.32
Accuracy	91.72	95.73	97.11

**Table 6:** Classification accuracy attained with various feature optimization algorithms in the OCI-LSTM, DNN and DBN.

Feature Optimization Algorithm	Optimal Features (Sign)	Accuracy of DNN (%)	Accuracy of DBN (%)	Accuracy of OCI-LSTM (%)
Correlation-based feature selection (CFS)	8(f_3, f_7 to f_13)	81.82	87.32	88.45
Chi-squared (Chi_Sq)	11 (f_1 to f_4 and f_7 to f_13)	87.88	90.17	91.46
Genetic Algorithm (GA)	8 (f_3, f_4, f_6 to f_10 and f_13)	81.14	92.62	94.51
Lease absolute shrinkage and selection operator (LASSO)	8 (f_2, f_3, f_5, f_8, f_9, f_11 to f_13)	84.51	91.46	94.7
Ruzzo–Tompa (RT)	7 (f_3,f_7 to f_10, f_12 and f_13)	90.57	94.61	96.82
SSA	7(f_1, f_7, f_8, f_9, f_10, f_12 and f_13)	91.72	95.73	97.11

## Data Availability

Online UCI Machine Learning Repository.
